# 

**Published:** 1887-05-07

**Authors:** 


### THE HOSPITAL

An Institution, Family, and Parochial Journal of Hospitals, Asylums, & all Agencies for the Care of the Sick, Criticism & News.

### CONTENTS.

**Fever Nurses: their Dangers and Defences** - 85

**Words of Consolation.**—XXXII. The Work of the Holy Ghost - - - - - - - 86

**Nurse Marion's Romance.** By KATE FURLEY. *Conclusion* - - - - - - - 87

**A National Pension Fund for Hospital Officials and Nurses.** The Views of Officials, Sisters, Nurses, and Others - - - - 88

**Insanity in Child-bearing.** By HENRY RAYNER, M.D., Medical Superintendent, Hanwell Asylum. Part I. Puerperal Forms - - - - - - 89

**Some Dangers of Spring.** By a FAMILY DOCTOR 90

**Chats about Medical Museums.** By ONE OF THE CROWD.—No. IV. University College - - - 90

**Notes and News.**—The Prince and Princess of Wales and the London Hospital.—Gifts to Hospitals.—The Islington Dispensary.—"First Aid."—Hospital Appeals.—Vacancies.—The British Ophthalmic Hospital, etc. - 92

**Annotations.** — The Hospital Porter. — Glasgow Charities and Doctors - - - - - 95

**Shall we give up the Novel?** By a COTTAGE HOSPITAL NURSE - - - - - - 95

**Reminiscences of the Insane.** By a MENTAL PHYSICIAN - - - - - - - 96

**Natural and Artificial Ventilation of Hospitals.** By C. THEODORE WILLIAMS, M.A., M.D., F.R.C.P. - - - - - - - 97

**Curiosities of Ancient Leechcraft.** By a STUDENT OF OLD BOOKS AND MANUSCRIPTS - - 98

**In- and Out-Patients' Hospital Diary** - - 99

**The Children's Corner.**—Puzzles, Answers, etc. - vi

**Amusements with Profit** - - - vi

